# Yttrium: A Highly Efficient Dopant for Ferroelectric HfO_2_

**DOI:** 10.1021/acsaelm.5c00936

**Published:** 2025-07-11

**Authors:** Mehrdad Ghiasabadi Farahani, César Magén, Alberto Quintana, Ignasi Fina, Florencio Sánchez

**Affiliations:** † Institut de Ciència de Materials de Barcelona (ICMAB-CSIC), Campus UAB, 08193 Bellaterra, Spain; ‡ Instituto de Nanociencia y Materiales de Aragón (INMA), CSIC-Universidad de Zaragoza, 50009 Zaragoza, Spain

**Keywords:** ferroelectric HfO_2_, epitaxial HfO_2_, doping, yttrium, ferroelectric oxides

## Abstract

The ferroelectric phase of HfO_2_ is metastable, and its stabilization in thin films strongly depends on both doping and microstructure. Optimally doped films exhibit high ferroelectric polarization; however, it typically decreases rapidly with increasing film thickness. In contrast to these usual results, a few exceptions report ferroelectricity in thick films with specific dopants, such as Y or La, crystallized under conditions that favor granular microstructure. Disentangling the roles of microstructure and doping on the thickness-dependent stability of ferroelectricity remains essential, and identifying highly effective dopant atoms is of high relevance. In this work, Y-doped epitaxial films of various thicknesses up to about 100 nm are prepared to determine phase evolution and ferroelectric polarization. Films deposited on SrTiO_3_(001) and SrTiO_3_(110) substrates exhibit robust ferroelectric response across the entire thickness range, in contrast to equivalent La-doped films. Coexistence of monoclinic (paraelectric) and orthorhombic (ferroelectric) phases is observed, with columnar grains revealed by scanning transmission electron microscopy, demonstrating that the microstructure of epitaxial HfO_2_ films can be preserved beyond 10 nm. The observed columnar grain structure indicates that the robustness of ferroelectricity in Y-doped films results from the high effectiveness of Y, supporting its use in devices requiring thick ferroelectric layers.

## Graphical abstract

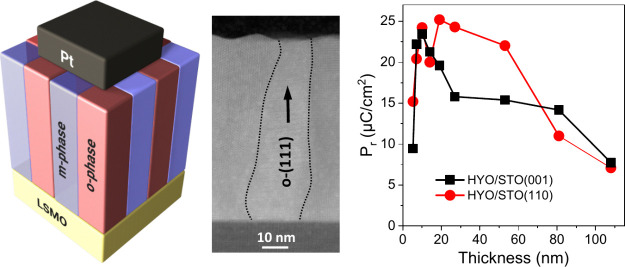

## Introduction

The discovery of ferroelectricity in HfO_2_,^1^ a material compatible with complementary metal-oxide semiconductor (CMOS) technology, has raised great expectations. This breakthrough discovery has opened new avenues for integrating ferroelectrics into nonvolatile memory devices.^2^ The ferroelectric properties of HfO_2_ films are attributed to the metastable polar orthorhombic (o) phase. This phase can be stabilized in ultrathin films through the introduction of a wide variety of dopant atoms.^2,3^ The o-phase generally coexists with other polymorphs, particularly with the nonferroelectric monoclinic (m) phase. The monoclinic phase is the most stable and it is usually predominant in films thicker than about 10–20 nm, causing a strong reduction of the ferroelectric polarization.^4–6^ This indicates that surface and interface energies critically influence the stabilization of the ferroelectric phase.^7^ In brief, the fraction of the stabilized orthorhombic phase in the film and the ferroelectric polarization depend on the specific chemical composition of the doped HfO_2_ (dopant atom and concentration) and its thickness.^2,3^ Experimental studies^8^ suggest that the content of orthorhombic phase and the polarization is higher when HfO_2_ is doped with atoms possessing a large ionic radius, such as Y, La, Gd or Sr, instead of the commonly used Zr. Theoretical calculations indicate that dopant atoms with larger ionic radius and lower electronegativity than Hf favor the stabilization of the polar orthorhombic phase.^9^ This is of great relevance, since it would imply that films doped with these atoms should show a more stable ferroelectric order. Other authors considered the influence of trivalent doping atoms on different defects, and comparing Al, Y, and La, they found the latter to be the most effective in stabilizing the ferroelectric phase.^10^ Despite extensive studies on the impact of the dopant on the formation of the metastable ferroelectric phase, a complete understanding of the factors promoting its stabilization has not yet been achieved.^11^

Additionally, the orthorhombic phase stability can be enhanced in films having a small grain size due to the influence of surface energy, thus allowing for thicker films. This microstructure can be obtained by using chemical solution deposition (CSD) instead of the commonly used atomic layer deposition (ALD), as it results in smaller grain sizes. A remanent polarization (*P*_r_) of 8 μC/cm^2^ was reported^12^ in *t* = 390 nm ZrO_2_ films grown by CSD. In contrast, the polarization of ZrO_2_ films prepared by pulsed laser deposition (PLD) or sputtering decreases sharply with thickness.^13–15^ CSD was also used^16^ to grow *t* = 1 μm La:HfO_2_ films, which showed a microstructure of grains of about 10 nm in size. This small grain size was likely determinant on the stabilization of orthorhombic phase as main phase, leading to a *P*_r_ ∼ 9 μC/cm^2^ despite the very high thickness. In contrast, epitaxial La:HfO_2_ films prepared by PLD show strong reduction of polarization in films thicker than around 10 nm.^17^ In the case of polycrystalline Y:HfO_2_ (HYO) films, prepared by CSD and with thickness in the 18–70 nm range, it was reported that the remanent polarization does not depend on thickness.^18^ Likewise, HYO films deposited by PLD at room temperature and crystallized by annealing have similar *P*_r_ in the investigated range of thickness from 10 nm to close to 1 μm.^19,20^ Analogous results were obtained by sputtering without neither intentional heating (only the inherent to the sputtering process) nor postannealing.^21^ Previously, the same authors had performed in situ X-ray diffraction (XRD) measurements and found that the tetragonal phase was much more stable at high temperature in HYO than in HfO_2_ films doped with other atoms.^22^ Based on this result, it was concluded that the stabilization of the ferroelectric phase in HYO was mostly determined by the specific chemical composition (i.e., doping with Y and with optimal content) and not by surface energy.^19^ However, the stability of the ferroelectric phase in such thick layers could also be favored by a small grain size. Indeed, other authors have reported a sharp reduction of polarization with thickness above about 10 nm in HYO films crystallized epitaxially in situ during the PLD process at high substrate temperature.^23^ These latter results suggest that, without surface energy contribution due to small-grain microstructure, the nonferroelectric monoclinic phase is dominant in HYO films thicker than about 10 nm. On the other hand, ferroelectricity has been confirmed in HYO single crystals.^24^ While this might suggest that Y is a highly effective dopant, stabilization of the ferroelectric phase was achieved in single crystals grown using laser radiation, which allowed for extremely high cooling rates. It was proposed that the high chemical pressure due to yttrium supersaturation allowed the formation of the orthorhombic phase,^24^ while a recent study suggests that pressure alone does not allow the formation of the orthorhombic phase.^25^ Further studies comparing single crystals with different dopants, or involving single crystals prepared by other techniques, may be very useful to gain more information. Overall, the higher robustness of HfO_2_ doped with some dopants, particularly Y, is unclear. Aiming to disclose its origin, we have prepared a series of epitaxial HYO films with a wide range of thicknesses and performed a thorough characterization of the structural and ferroelectric properties to understand the role of Y as a dopant. In order to select the most efficient yttrium content to stabilize the ferroelectric phase, we considered the optimal Y concentration that maximizes the orthorhombic phase fraction and polarization in precedent works, which is dependent on the used deposition technique, as observed for La doped films.^26^ It is reported that in Y-doped HfO_2_ films prepared by ALD, the highest polarization is obtained with an atomic concentration in the range of 3–5%.^27^ Using sputtering the optimal yttrium concentration reported is 1–2%.^28^ In contrast, studies of the effect of Y concentration on PLD-prepared layers reveal an optimum content of 7%.^29,30^ Yttrium concentration of 7% is also used in other studies to obtain high polarization films by PLD.^31,32^ Therefore, we selected the Y concentration of 7% (Hf_0.93_Y_0.07_O_2_) to prepare a series of HYO epitaxial films with thicknesses in the range of 3–110 nm on (001)- and (110)-oriented SrTiO_3_ (STO) substrates. We have performed a comprehensive structural and electrical characterization of the HYO films that confirms that these are all ferroelectric having high polarization in the whole investigated thickness range. In contrast, the equivalent epitaxial films of HfO_2_ doped with Zr and/or La and deposited using the same conditions showed a strong reduction of orthorhombic phase content and polarization for thicknesses higher than about 10 nm.^17,33–35^ We show that the columnar grain microstructure is preserved even for thick Y-doped films. Thus, our work demonstrates that Y is an inherently much more efficient dopant than the more commonly used Zr and La to stabilize the metastable ferroelectric phase of HfO_2_, and disregards grain size effects as a main mechanism for the stabilization of ferroelectricity in thick films.

## Experimental Section

HYO films and bottom La_0.67_Sr_0.33_MnO_3_ (LSMO) electrodes were grown in a single process using pulsed laser deposition with a KrF excimer laser. Sintered Hf_0.93_Y_0.07_O_2_ and La_0.67_Sr_0.33_MnO_3_ ceramics were used as targets. The cations of both compounds are not particularly volatile, allowing the cationic composition of each target to be replicated in the deposited LSMO and HYO films. The LSMO electrodes were deposited at a substrate temperature of 700 °C, an oxygen pressure of 0.1 mbar, and a laser frequency of 5 Hz on STO(001) and STO(110) substrates. The STO substrates were used as received, without undergoing any chemical treatment. For the growth of HYO films, the corresponding parameters were 2 Hz, 800 °C, and 0.1 mbar. Two series of HYO films with thicknesses in the 3.3–108 nm range, deposited on LSMO buffered STO(001) and STO(110) substrates, were prepared. Circular platinum top electrodes, 20 μm in diameter and 20 nm in thickness, were grown ex-situ by sputtering onto the HYO films through stencil masks for electrical measurements. Crystal structure analysis was performed using XRD with Cu K_α_ radiation employing a Siemens D5000 diffractometer equipped with a point detector and a Bruker D8-Advance diffractometer equipped with a 2D detector. Atomic scale structural analysis was conducted by aberration corrected scanning transmission electron microscopy (STEM) imaging in high-angle annular dark field (HAADF) mode. STEM images were collected in a Thermo Fisher Scientific Titan Low Base 60–300 equipped with a CETCOR aberration corrector for the condenser system and a high brightness Schottky field emission gun (X-FEG), with an electron probe size below 1 Å. Ferroelectric polarization loops were measured at room temperature using an AixACCT TFAnalyser3000 platform, with the LSMO bottom electrode connected to the ground and bias applied to the top Pt contact. Ferroelectric polarization loops were obtained in dynamic leakage current compensation (DLCC) mode with a frequency of 1 kHz. The residual leakage contribution was removed by compensation processing (Figure S1).

## Results and Discussion

XRD 2θ–χ maps of the series of films on STO(001) and STO(110) are shown in Figure 1a,b, respectively. In addition to the high-intensity (001) and (002) (Figure 1a) and (110) (Figure 1b) reflections arising from the substrate and the LSMO electrode, there are also spots corresponding to the HYO film in all the samples. The bright circular spot at χ = 0° and 2θ ∼ 30°, the position of the o(111) reflection, increases in intensity with thickness. In addition, a spot corresponding to the m­( 1̅11 ) reflection is present for films thicker than 50 nm. The elongation of this spot along χ indicates mosaicity of the monoclinic crystallites with (1̅11) texture (Figure S2). Weak spots corresponding to the position of the m{002} reflections can also be observed in the films thicker than ∼10 nm on the STO(001) substrate, while overlapping with the STO(110) reflection in the films on this substrate, thus challenging to determine its presence. Figure 1c,d shows θ–2θ scans measured with a point detector around the main reflections of the HYO films on both substrates. The o(111) peak is accompanied by Laue oscillations and it is broader as the film is thinner due to the size effect. The m(1̅11) peak at 2θ ∼ 28.5° is observed in films thicker than 50 nm but not in thinner ones and, consistent to the XRD 2θ–χ maps shown in Figure 1a, m{002} reflections are also observed in films thicker than ∼20 nm on STO(001) (Figure 1c).

**Figure 1:**
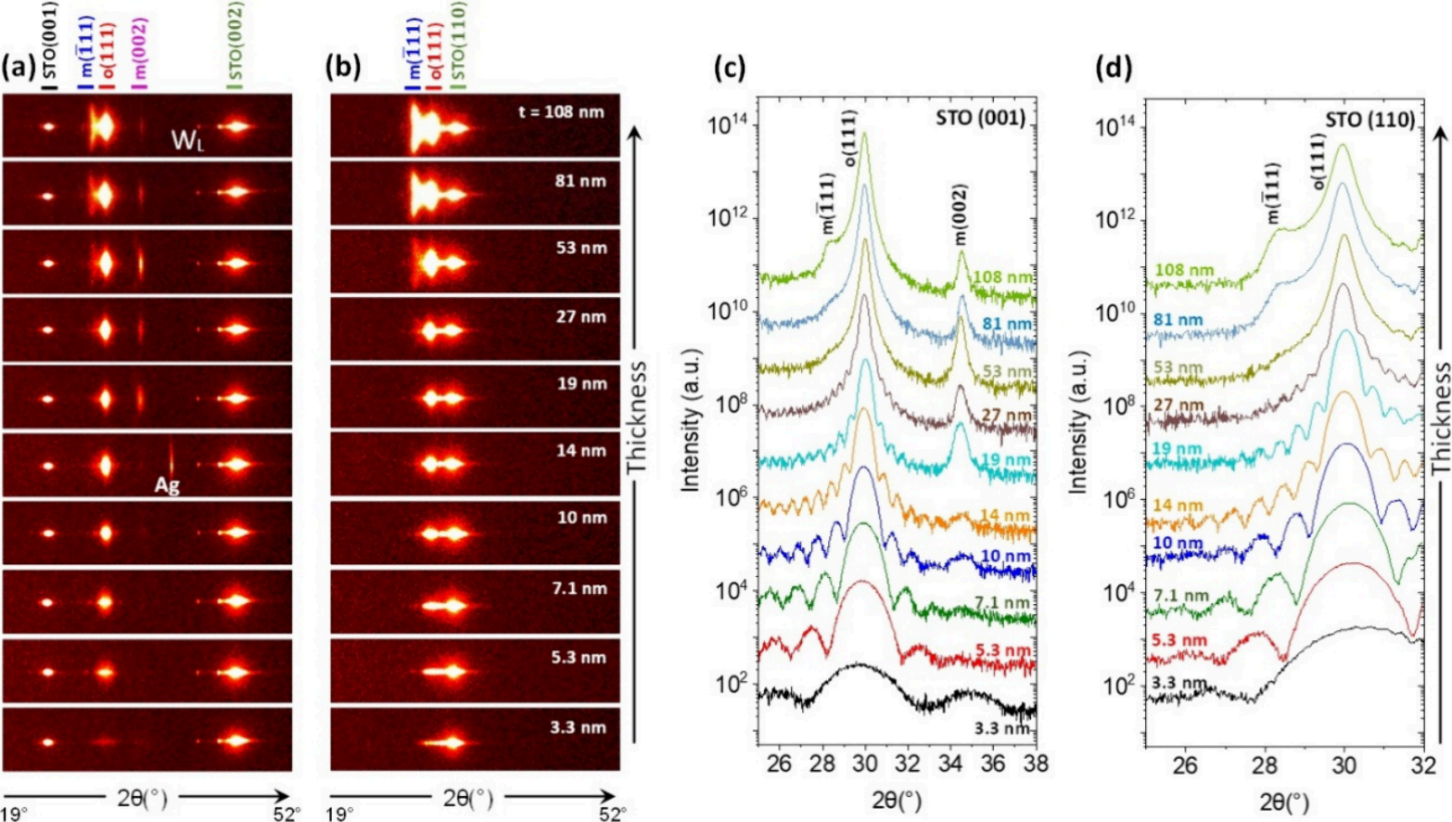
XRD 2θ–χ frames of the films on (a) STO(001) and (b) STO(110). The STO(001) and (002) peaks are indicated by black and green lines, respectively. The o(111), m(−111), and m(002) HYO reflections are marked with red, blue, and pink lines, respectively. In panel (a), the spot at ∼42° is the STO(002) reflection due to the spurious X-ray W_L_ line, and the weak reflection at 38° in the *t* ∼ 15 nm film on STO(001) is due to the silver used for electrical measurements. (c) and (d) XRD θ–2θ scans, measured with a point detector, of films on STO(001) and STO(110), respectively.

The intensities of the peaks from θ–2θ scans (Figure S3) obtained by integration through χ from −10° to +10° in the 2θ–χ maps (Figure 1a,b) are shown in Figure 2a. The intensity of the o(111) peak increases with thickness and the observed trend is very similar for films on both substrates. The m(002) peak is not observed in any film on STO(110), and it is only evident in films thicker than ∼20 nm on STO(001). The m(002) peak intensity does not exhibit a clear dependence thickness, with the most intense m(002) peak in the *t* = 53 nm film and with much lower intensity in the thicker films. The m(1̅11) peak, which does not overlap with substrate reflections, is evident in films thicker than ∼50 nm on both substrates, increasing in intensity with thickness, and being significantly more intense in films on STO(110) than in the equivalent films on STO(001). These data, calculated from the measurements performed with a 2D detector, are in good agreement with the ones extracted from the θ–2θ scans in Figure 1c,d (Figure S4).

**Figure 2:**
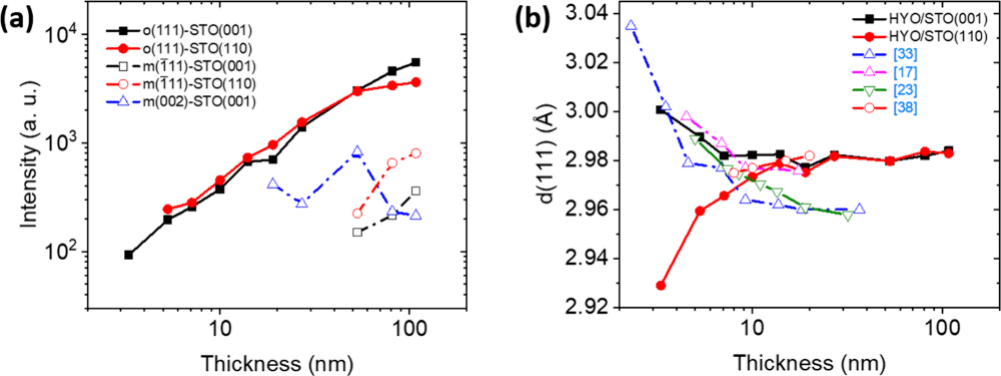
(a) Thickness dependence of o(111), m(1–11), and m(002) peak intensities. (b) Thickness dependence of the out-of-plane lattice parameter *d*_o(111)_. The figure includes data corresponding to other epitaxial films of doped HfO_2_ films reported in the literature: La:HfO_2_/STO­(001);^17^ HYO/STO(001),^23^ HZO/STO(001),^33^ and La:HfO_2_/STO­(110).^38^

In the measurements with point detector, the higher intensity of the o(111) reflections of films on STO(110) compared with films of the same thickness on STO(001) is due to their lower mosaicity (see the χ-scans in Figure S5) and not to a larger amount of the o-phase. On the other hand, the out-of-plane lattice parameter, *d*_o(111)_, was determined from the position of the o(111) diffraction peak in the θ–2θ scans shown in Figure 1c,d. The *d*_o(111)_ value of films on STO(001) is around 3.0 Å for the *t* = 3.3 nm film and decreases to less than 2.98 Å for the *t* = 19 nm film, remaining this value constant for thicker films (Figure 2b). In contrast, the *d*_o(111)_ of films on STO(110) is only 2.93 Å for the *t* = 3.3 nm film and it increases with thickness and stabilizes at around 2.98 Å for around 20 nm. Figure 2b also includes *d*_o(111)_ values reported in literature for epitaxial doped HfO_2_ films on (001) and (110) oriented substrates. A decrease of *d*_o(111)_ with thickness is recurrently found in epitaxial films on STO(001).^17,23,33,34,36^ On the other hand, the thickness dependence of *d*_o(111)_ of films on (110)-oriented substrates has been less investigated, but it is reported that in ultrathin Hf_0.5_Zr_0.5_O_2_ (HZO) films on STO(110) *d*_o(111)_ is smaller than in equivalent films on STO(001)^37^ and reported data^38,39^ suggest for HZO films on STO(110) an increase of *d*_o(111)_ with thickness similar to that of our HYO films on STO(110).

The leakage current curves of the films are shown in Figure S6. The films thicker than 5 nm have low leakage of about 10^–7^–10^–6^ A/cm^2^ at 1 V, without dependence on film thickness or substrate orientation. However, leakage is much higher in the thinnest films, *t* = 3.3 nm, which precluded polarization measurements. The high leakage of the ultrathin *t* = 3.3 nm films is likely due to the higher probability of pinholes and to the presence of tunneling current expected for this thickness. The polarization loops of all the HYO films thicker than 5 nm on STO(001) are shown in Figure 3a. The leakage contribution in all polarization loops was removed (Figure S7 shows the loops before and after leakage contribution subtraction). The films do not exhibit wake-up effect in measurements under high electric field (Figure S8). Remarkably, the remanent polarization of the *t* = 5.3 nm film is about 10 μC/cm^2^, a high value considering the low thickness. Moreover, Figure 3a shows that HYO films on STO(001), with thicknesses up to about 100 nm are ferroelectric. The remanent polarization first increases with thickness up to a maximum of *P*_r_ ∼ 33.5 μC/cm^2^ at 10 nm, and decreases in thicker films to *P*_r_ ∼ 14 μC/cm^2^ in the *t* = 81 nm film and about 8 μC/cm^2^ in the *t* = 108 nm film (Figure 4a). The reduction of polarization in the thicker films is due to the increased fraction of monoclinic phase and to undersaturation conditions (the application of a larger electric field is not possible since the maximum output voltage of the setup is 35 V). The ferroelectric loops of the HYO film series on STO(110) exhibit a similar thickness dependence (Figure 3b), albeit with high remanent polarization over a wider thickness range (Figure 4a). The wake-up effect is also very small (Figure S9)). On the other hand, HYO films thinner than about 10 nm on both STO(001) and STO(110) substrates show the same thickness dependence, and for same thickness the polarization values are similar. This sharply contrasts with the opposite dependence of strain on layer thickness on both substrates (Figure 2b). The different dependence of *P*_r_ and strain on thickness indicates the reduced effect of strain on the stabilization of the ferroelectric phase in hafnia and, thus on its polarization.^40,41^

**Figure 3:**
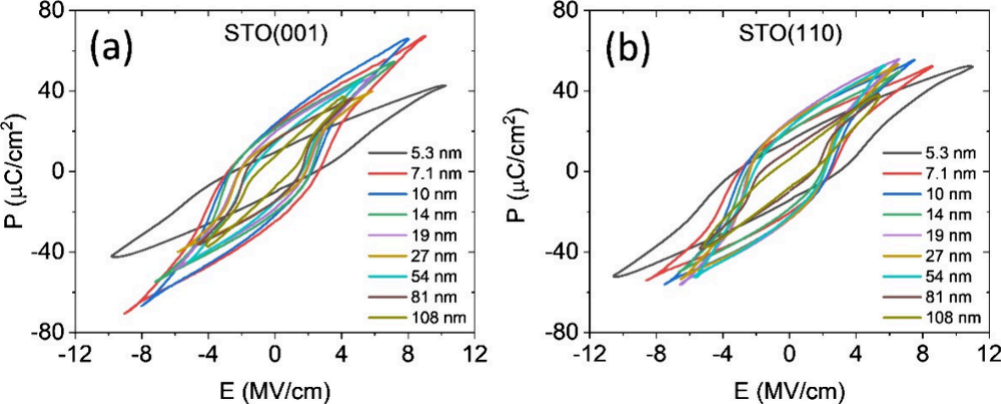
Polarization loops for films on (a) STO(001) and (b) STO(110).

**Figure 4:**
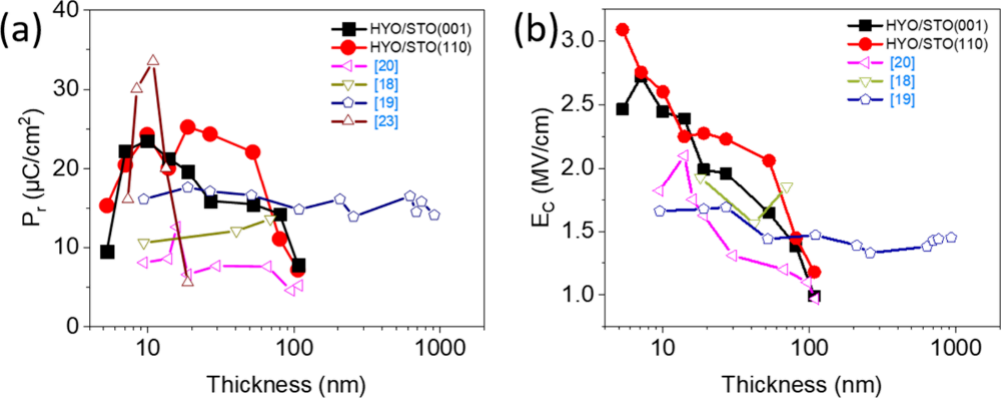
(a) Solid symbols: remanent polarization as a function of the thickness for films on STO(001) (black squares) and STO(110) (red circles). (b) Coercive field as a function of the thickness of films on STO(001) (black squares) and STO(110) (red circles). Open symbols in panels (a) and (b) correspond to data reported in the literature for epitaxial^20,23^ and polycrystalline^18,19^ HYO films.

The thickness dependence of the coercive electric field (*E*_C_) (Figure 4b) is similar for both series of films on STO(001) and STO(110). *E*_C_ decreases with thickness from ∼3 MV/cm (*t* ∼ 5 nm) to ∼1.2 MV/cm (*t* = 108 nm). Therefore, contrary to the usual lack of thickness dependence of the coercive field for polycrystalline doped HfO_2_ films,^42–44^ the HYO epitaxial films exhibit a significant decrease of *E*_C_ with thickness. However, the reduction is smaller (slope is ∼0.27) than in equivalent epitaxial films doped with other atoms, for which the dependence is close to *E*_C_ ∝*t* ^–2/3^.^17,33,34^ Figure 4b also shows the *E*_C_ values reported for polycrystalline^18,19^ and epitaxial^20^ films prepared by solid-phase epitaxy. The coercive field of the polycrystalline films^18,19^ does not depend on the thickness, while the *E*_C_ for the reported epitaxial samples^20^ exhibits a reduction with thickness similar to the epitaxial HYO films on STO(001) and STO(110).

The robustness of ferroelectricity in Y-doped epitaxial HfO_2_ films upon increasing thickness differs with respect to the strong polarization loss with thickness in epitaxial HZO,^33^ La-doped HZO^34^ and La-doped HfO_2_ ^17^ films prepared under the same deposition conditions (Figure 5). Figure 4a, where the thickness dependence of the remanent polarization of HYO epitaxial films on STO(001) and STO(110) is plotted, also includes data reported in the literature for HYO. The ferroelectric phase in films tens of nanometers thick is highly stable in polycrystalline HYO films prepared by CSD^18^ (gold open triangles facing down) or by PLD at room temperature and annealing^19^ (blue open pentagons). A similar lack of thickness dependence^20^ was observed for epitaxial HYO films prepared by solid-phase epitaxy (magenta open triangles), while for films epitaxially grown by conventional PLD (brown open triangles), disappearance of polarization for films thicker than about 20 nm was reported.^23^ In contrast, our HYO films, also epitaxial and prepared by conventional PLD, remain ferroelectric in the whole thickness range investigated, up to about 100 nm. The differences in the robustness of ferroelectricity increasing HYO film thickness could be caused by differences in the microstructure, and particularly because high granularity, could favor the stability of the orthorhombic phase. Indeed, the fact that the coercive electric field decreases with thickness, but with smoother dependence than *E*_C_ *∝t* ^–2/3^ , does not conclusively indicate that granularity effects are ruled out. Therefore, we have performed STEM characterization to discern whether the persistence of ferroelectricity in HYO films tens of nm thick is a consequence of high efficiency of Y as a doping atom or is caused by high granularity due to the deposition technique used or the particular deposition conditions.

**Figure 5:**
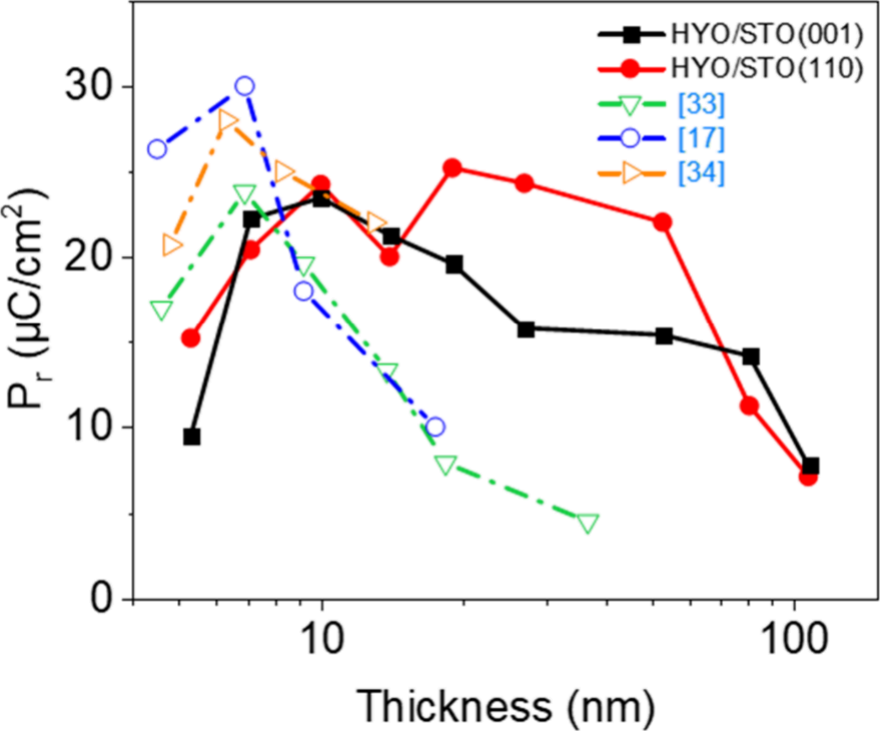
Dependence on thickness of the remanent polarization of films on STO(001) (solid black squares) an STO(110) (solid red circles). Empty symbols correspond to equivalent epitaxial films doped with other atoms and that we reported elsewhere: HZO (green triangles),^33^ La:HfO_2_ (blue circles),^17^ and La:HZO (orange triangles).^34^

Figure 6a,d depicts low magnification HAADF-STEM images obtained from 80 nm-thick HYO films grown on STO(100) and STO(110). Diffraction contrast, which is particularly strong in the films grown on STO(100), evidence that the films present a columnar microstructure, in which it is easy to find elongated grains extending through the whole film thickness. This microstructure is similar to the usually found in epitaxial films of HZO, deposited under same conditions, but thinner than 10 nm.^37,45,46^ While a quantitative study of the presence of crystal phases and possible textures present in the epitaxial HYO films on STO(001) and STO(110) is beyond the scope of this work, there is an abundance of orthorhombic grains with o(111) texture in both films; examples of these are shown in Figure 6b,e, which have been indexed in the fast Fourier transforms (FFT) shown in the insets. Fewer monoclinic grains can be identified, for instance, m(002) and m(−111)-oriented crystals are depicted in Figure 6c,f, respectively. These results support that the adequate Y-doping promotes the growth of HYO films with columnar microstructure which enables the stabilization of a majority orthorhombic phase with strong ferroelectric properties even in thick Y-doped HfO_2_ films up to 100 nm. The fact that this occurs in thick films with large (tens of nanometers in size) elongated grains suggests that optimal doping with Y can play a dominant role over grain surface energy in the stabilization of the ferroelectric phases of HfO_2_.

**Figure 6:**
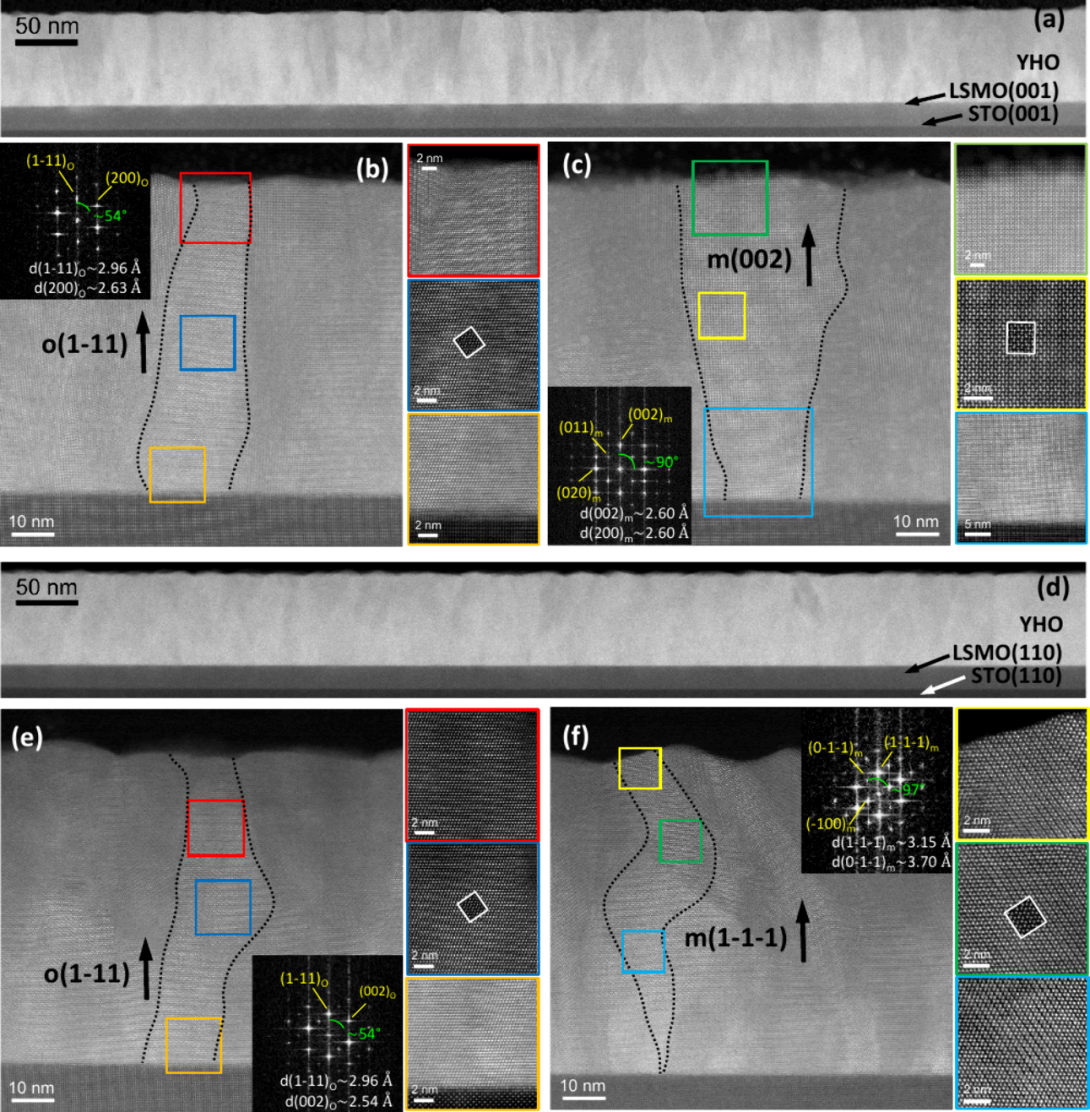
Low-magnification HAADF-STEM image of the 80 nm-thick HYO film on STO(001) (a) and STO(110) (d). High-resolution HAADF-STEM images of these films grown on STO(001) (b, c) and STO(110) (e, f) showing examples of columnar grains crystallizing in the orthorhombic and monoclinic phases. Dashed line boundaries are a guide to the eye. Color-framed pictures correspond to atomic-resolution images collected from the regions marked with the respective colored squares in panels (b, c) and (e, f). The white framed insets are the simulated STEM image of the proposed structure. Indexed FFT images of the central areas of the highlighted grains are shown in the insets of panels (b), (c), (e), and (f).

## Conclusions

In summary, Y-doped HfO_2_ epitaxial films of thickness about 100 nm have robust ferroelectricity and transmission electron microscopy confirms that orthorhombic grains are columnar and extend along the entire film thickness. This is the typical microstructure of HfO_2_ epitaxial films, doped with common dopant atoms such as Zr or La. But unlike equivalent Zr- or La-doped HfO_2_ epitaxial films, the robust ferroelectricity of Y-doped HfO_2_ does not vanish with thickness. This demonstrates that Y is inherently a more efficient dopant atom to stabilize the ferroelectric phase of hafnia and that not only size effects are at play. Y-doped HfO_2_ films could be advantageous over films of hafnia doped with other atoms for some applications, particularly those requiring thick or relatively thick films.

## Supplementary Material



## Notes

XRD χ-scans around m(−111), m(−111), and m(002) reflections; θ–2θ scans obtained from measurements with a 2D detector; XRD χ-scans around o(111) reflection; dependence on thickness of the intensity of XRD reflections HYO; leakage current measurement; leakage compensation; ferroelectric polarization loops of selected films on STO(001) measured with different voltages and after a few cycles; and ferroelectric polarization loops of selected films on STO(110) measured with different voltages and after a few cycles (PDF)
