# Supplementary material for: Yttrium: A Highly Efficient Dopant for Ferroelectric HfO2
Source: ACS Appl Electron Mater. 2025 Jul 11;7(14):6628–34. doi: 10.1021/acsaelm.5c00936 (PMC12309089; doi:10.1021/acsaelm.5c00936)
Supplement: Supplementary file 1 [file el5c00936_si_001.pdf]

SUPPORTING INFORMATION

Yttrium: A highly efficient dopant for ferroelectric HfO_2

Mehrdad Ghiasabadi Farahani, [1] *César Magén,* [2] *Alberto Quintana,* [1] *Ignasi Fina,* [1,*] *Florencio Sánchez*[1,*]

[1] Institut de Ciència de Materials de Barcelona (ICMAB-CSIC), Campus UAB, Bellaterra 08193, Spain

[2] Instituto de Nanociencia y Materiales de Aragón (INMA), CSIC-Universidad de Zaragoza, 50009 Zaragoza, Spain

[*] Corresponding authors: ifina@icmab.es, fsanchez@icmab.es

Figure S1: leakage compensation in polarization loops

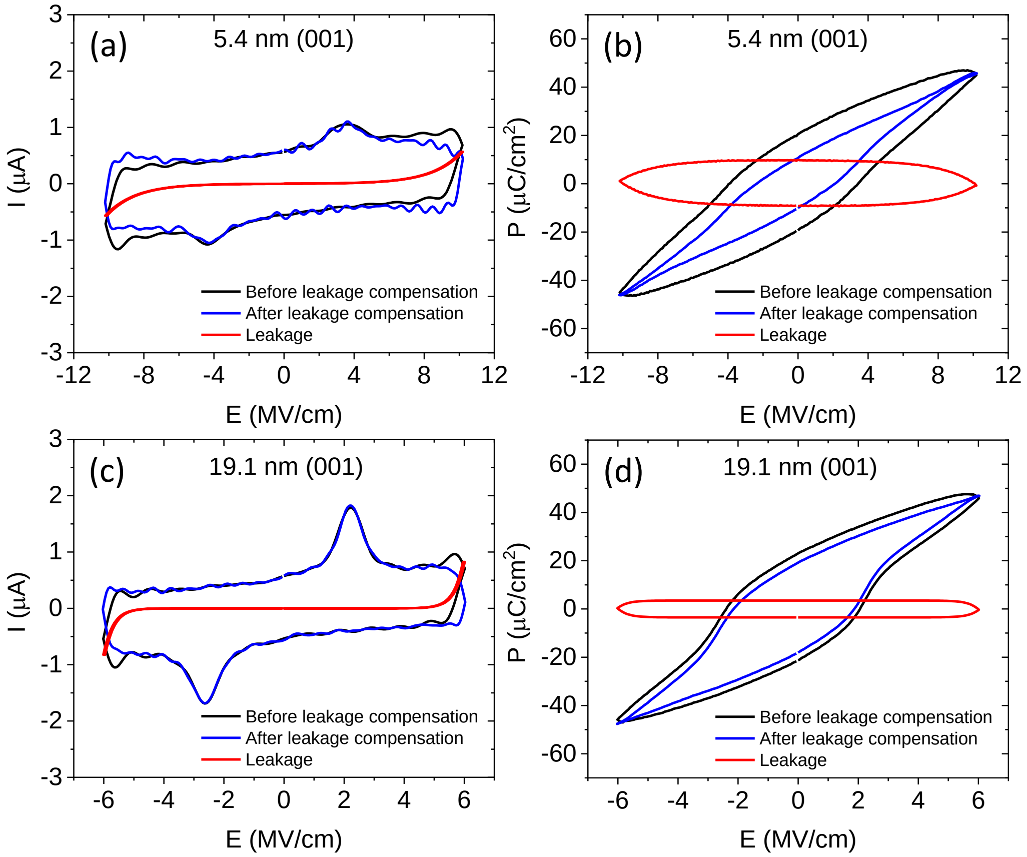

Figure S1. (a, b) I-E and P-E loops, respectively, before and after residual leakage compensation and also of the subtracted leakage contribution for 5.4 nm film on STO(001). (c, d) Corresponding I-E and P-E loops for 19.4 nm film on STO(001). It can be observed in Figures S1a that the current increase near the maximum applied electric field – attributed to leakage- is reduced after the compensation. Similarly, in Figure S1b, the round shape of the P-E loop near the maximum applied electric field is removed, resulting in a reduced and more accurate determination of polarization. Similar effect is shown in the 19.1 nm sample (Figure S1c and S1d), being here the removed leakage contribution smaller and therefore the impact on the shape of the P-E loop also smoothed, as expected by the reduced leakage in thicker films.

Figure S2: XRD χ-scans around m(-111), m(-111) and m(002) reflections

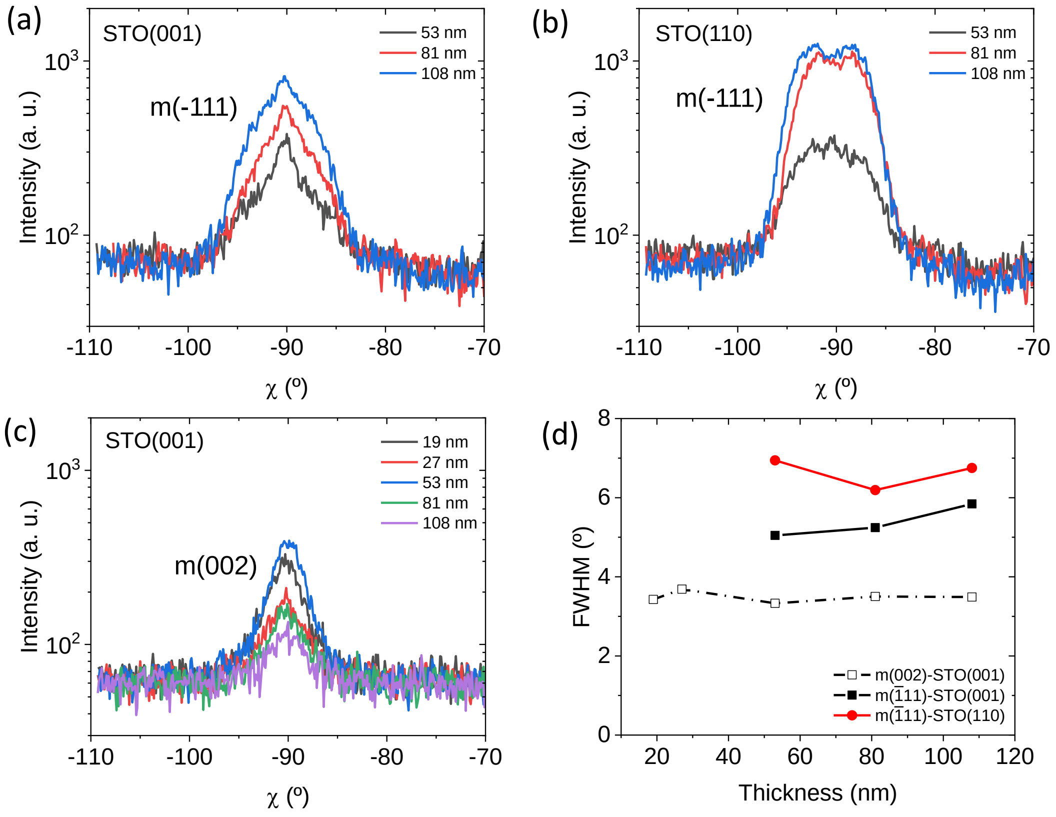

Figure S2. XRD χ-scans obtained by integration of the 2θ–χ maps around (a) m(-111) reflection of HYO on STO(001), (b) m(-111) reflection of HYO on STO(110), and (c) m(002) of HYO on STO(001). The 2θ integration ranges were 27°<2θ<28° and 34°<2θ<35° for m(-111) and m(002), respectively. (d) Full-width at half-maximum (FWHM) of the indicated χ-scans as a function of HYO thickness.

Figure S3: θ-2θ scans obtained from measurements with a 2D detector

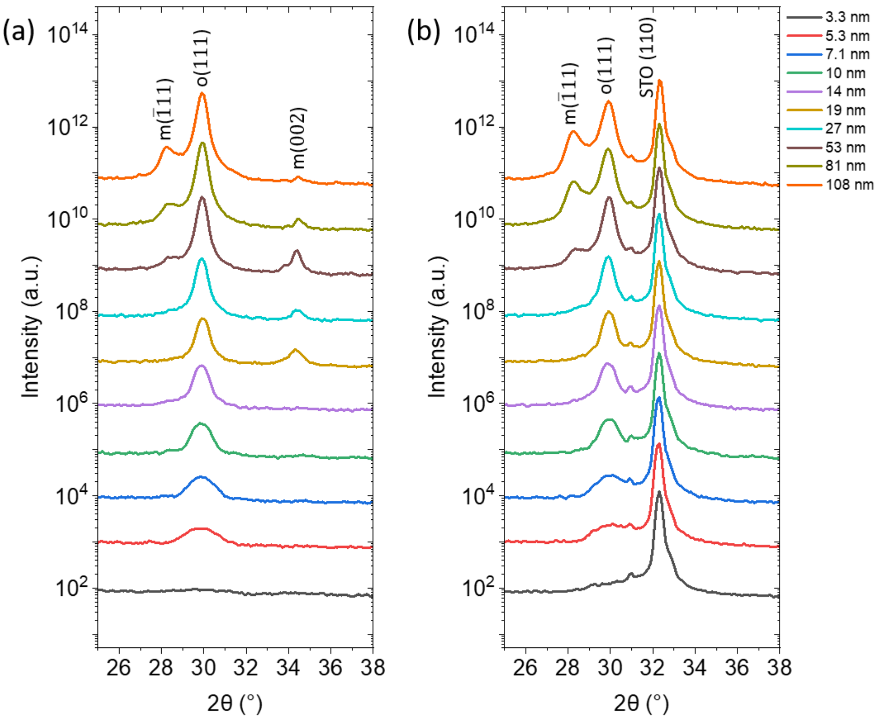

Figure S3. XRD θ-2θ scans obtained by integration in χ from −10° to +10° of the 2θ–χ maps shown in Figures 1a and 1b, respectively, corresponding to films on (a) STO(001) and (b) STO(110). In (b), the spot at ~31° is the STO(110) reflection due to the spurious X-ray W_L line.

Figure S4: Dependence on thickness of the intensity of XRD reflections HYO

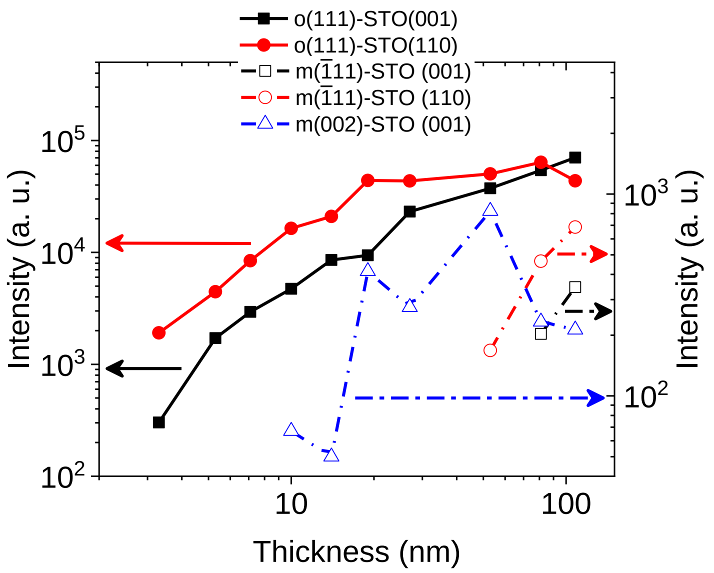

Figure S4. Thickness dependence of intensity of o(111), m($\bar{1}$11), m(002) reflections of XRD θ-2θ scans measured with point detector XRD. The corresponding scans for films on STO(001) and STO(110) are shown in Figures 1c and 1d, respectively.

Figure S5: XRD χ-scans around o(111) reflections

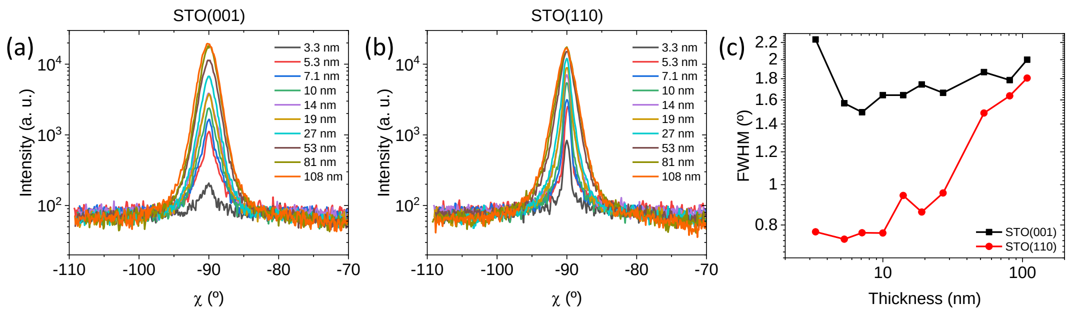

Figure S5: XRD χ-scans obtained by integration of the 2θ–χ maps around o(111) reflection of HYO on (a) STO(001) and (b) STO(110). The 2θ integration range was 29.5°<2θ<30.5°. (c) FWHM of the χ-scans around o(111) as a function of HYO thickness for films on STO(001) and STO(110).

Figure S6: Leakage current measurement

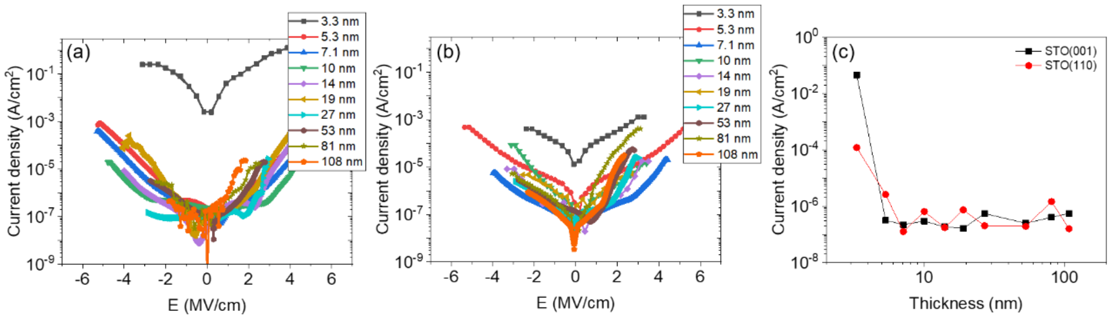

Figure S6. Leakage current curves of HYO films of the indicated thickness on (a) STO(001) and (b) STO(110). (c) Thickness dependence of the current leakage at 1 MV/cm (average value for positive and negative values) for films on STO(001) (black squares) and STO(110) (red circles).

Figure S7: Leakage compensation

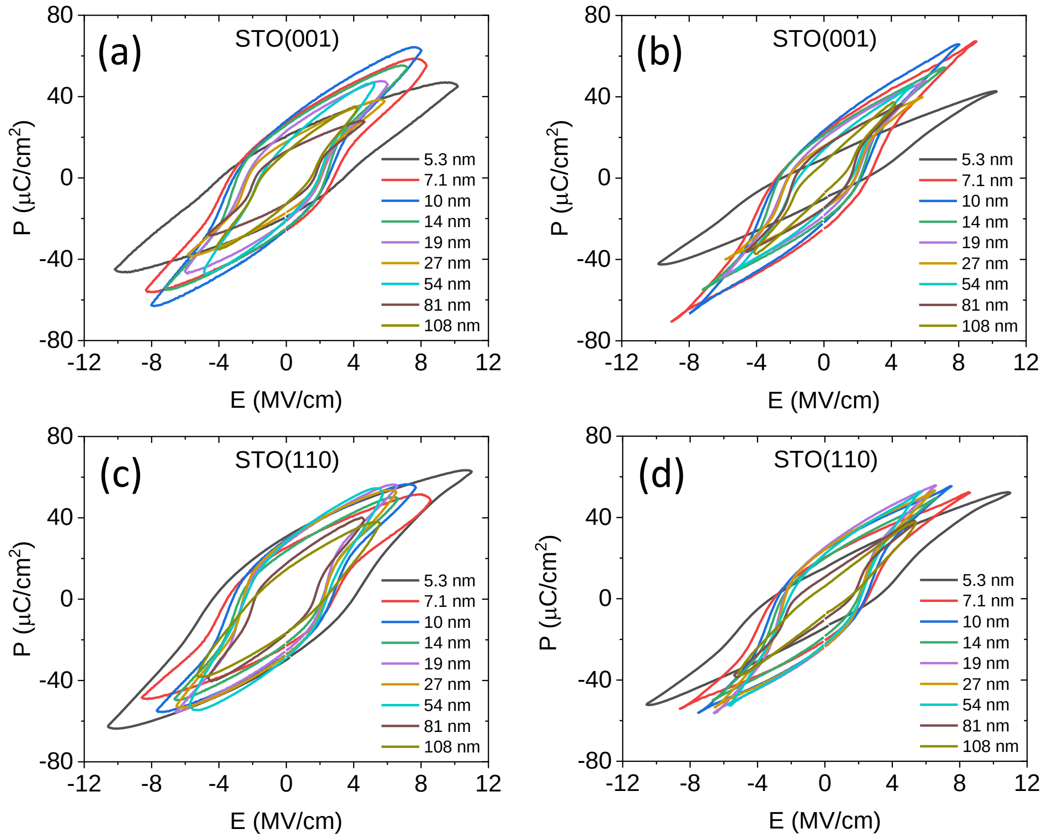

Figure S7. (a) and (b): Polarization (P) vs. electric field (E) loops for HYO films on STO(001), with (b) showing the corresponding loops after subtracting the leakage contribution. (c) and (d): P-E loops for HYO films on STO(110), with (d) also after leakage subtraction.

Figure S8: Ferroelectric polarization loops of selected films on STO(001) measured with different voltages and after a few cycles

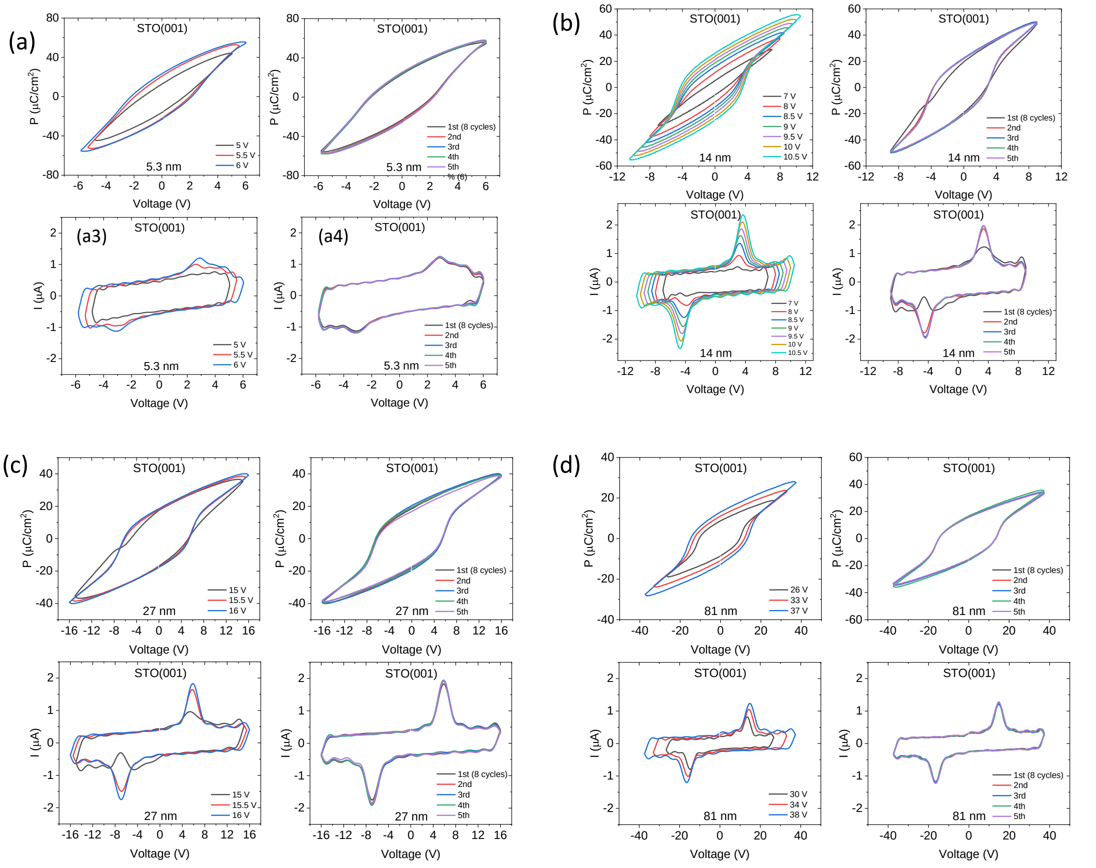

Figure S8. Polarization loops measured with varied maximum applied voltage and evolution with the number of cycles, and the corresponding current-voltage curves for (a) t = 5.3 nm, (b) t = 14 nm, (c) t = 27 nm, and (d) t = 81 nm films on STO(001).

Figure S9: Ferroelectric polarization loops of selected films on STO(110) measured with different voltages and after a few cycles

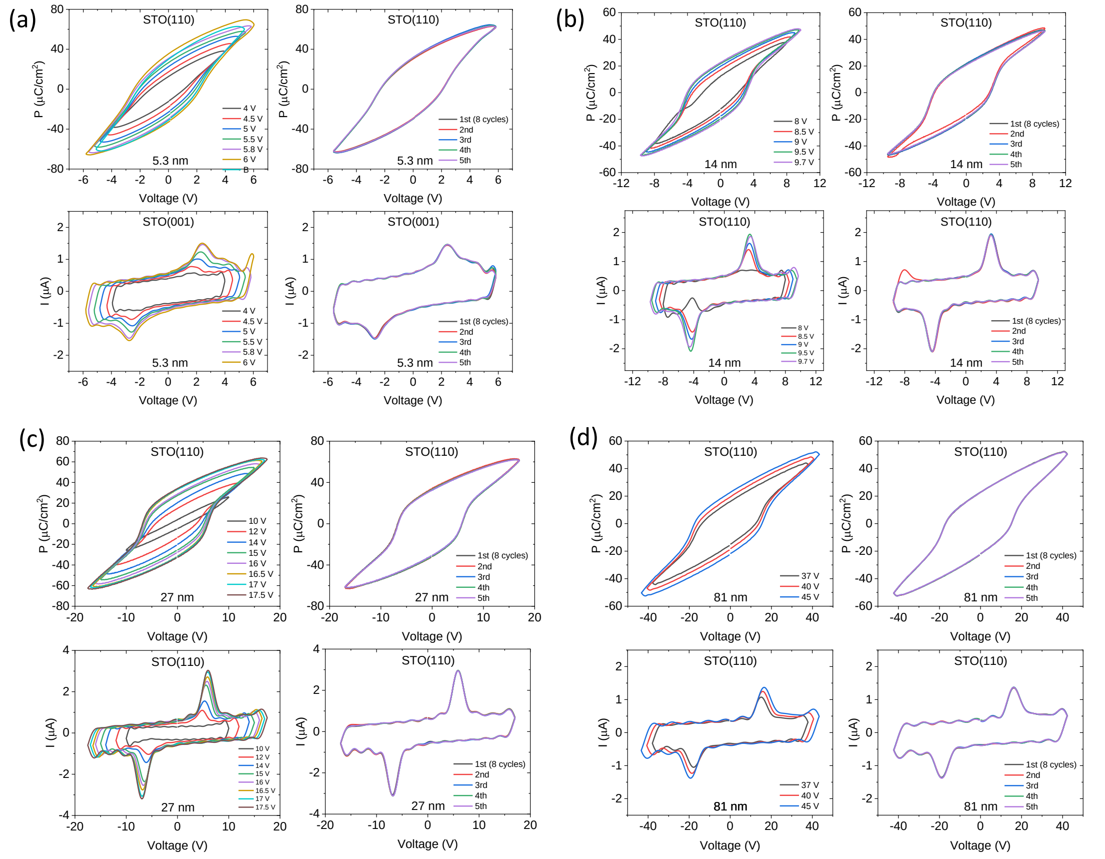

Figure S9. Polarization loops measured with varied maximum applied voltage and evolution with the number of cycles, and the corresponding current-voltage curves for (a) t = 5.3 nm, (b) t = 14 nm, (c) t = 27 nm, and (d) t = 81 nm films on STO(110).
